# Comparison of three different internal fixation implants in treatment of femoral neck fracture—a finite element analysis

**DOI:** 10.1186/s13018-019-1097-x

**Published:** 2019-03-12

**Authors:** Jia Li, Zhe Zhao, Pengbin Yin, Licheng Zhang, Peifu Tang

**Affiliations:** 1 Department of Orthopedics, Chinese PLA General Hospital, No. 28 Fuxing Road, Beijing, 100853 People’s Republic of China; 2 Department of Orthopaedics, Beijing Tsinghua Changgung Hospital, School of Clinical Medicine, Tsinghua University, No. 168, Li Tang Road, Changping District, Beijing, 102218 China

**Keywords:** Femoral neck fractures, Finite element analysis (FEA), Slide compression anatomic place-femoral neck (SCAP-FN), Derotational screw (DS)

## Abstract

**Background:**

Current surgical interventions for the femoral neck fracture are using either cannulated screws (CCS) or a single large screw at a fixed angle with a side-plate (i.e., a sliding hip screw, AKA dynamic hip screw, DHS). Despite these interventions, the need for reoperation remains high (10.0–48.8%) and largely unchanged over the past 30 years. Femoral neck fracture is associated with substantial morbidity, mortality, and costs.

**Methods:**

In this study, our group designed a plate that combines the strength of both CCS and sliding hip screw, through providing three dynamic screws at a fixed angle with a side-plate, namely the slide compression anatomic place-femoral neck (SCAP-FN). Finite element analyses (FEA) were carried out to compare the outcomes of the combination of our SCAP-FN plate with DHS+DS (derotational screw) and to those of using cannulated screws alone.

**Results:**

SCAP-FN produces more stable fixation with respect to the femur and the stress distributions, stress peaks, and rotational angles.

**Conclusions:**

The FEA encouraged us that in the following biomechanical experiment, SCAP-FN may remain the strengths of both CCS and DHS+DS and show a better performance in resisting shearing and rotational forces, therefore achieving the best stability in terms of smallest displacement and rotational angle.

**Electronic supplementary material:**

The online version of this article (10.1186/s13018-019-1097-x) contains supplementary material, which is available to authorized users.

## Introduction

Worldwide, approximately 1.5 million hip fractures occur annually, and this number is expected to increase to 6.3 million by 2050 [1]. The mortality rate is high (typically reported from 8.4 to 36% within 1 year) [1–3]. Despite surgical intervention, the reoperation rate remains high (10.0–48.8%), has remained largely unchanged, and is associated with substantial morbidity, mortality, and costs [4, 5]. The high proportion of reoperations has generated controversy about the optimum approach for fixing femoral neck fractures [6].

The mainstream surgical interventions currently used in clinical practice include cannulated screws or a single large screw at a fixed angle with a side-plate (i.e., a sliding hip screw, AKA dynamic hip screw, DHS). Multiple studies have compared the effectiveness of these approaches regarding reoperation rates and patient outcome. Biomechanical and laboratory studies suggest that although a sliding hip screw provides greater resistance to shearing force (the major cause of implant failure particularly in displaced and unstable fracture types), multiple cancellous screws (CCS) are less invasive and provide improved resistance to rotational forces (the second largest cause of implant failure) [7–9]. Previous trials including large sample-sized, internationally randomized controlled trials (RCTs) did not identify differences between the two fixation approaches according to patient outcomes, particularly the rate of reoperations, leaving uncertainty among surgeons as to the optimal approach for fixing femoral neck fractures. Of note, reoperation rates for both cannulated screws and DHS groups remain high (≥ 20%), and as such, clinicians continue to explore the next generation of effective fixation implants [10].

Knowledge of the pros and cons of each implant from biomechanical and laboratory studies has given us an inspiration to design a new implant for treatment of hip fracture. Shearing and rotational forces require resistance to achieve stability. Our design combines the strength of both CCS and sliding hip screw by providing three dynamic screws at a fixed angle with a side-plate, namely slide compression anatomic place-femoral neck (SCAP-FN). The plate was a pre-contoured plate. The surface of the plate was designed to fit the morphology of the proximal femur, and the distribution of the three screws was also considered the geometrical morphology of the femoral neck. The data set we used included over 400 Chinese femurs, it was also used in our prior work. The angle between screws and plate was designed to fit the average of Chinese population. The neck shaft angle of Chinese femur was about 122° on average, and the angle between screws and plate was designed based on this data. Due to the screw of SCAP-FN could provide sliding after surgery, the interface of the plate and screw was designed as a locking mechanism, which could have better angular stability.

Here in this study, we performed finite element analysis to compare the three implants in treating unstable femoral neck fracture with respect to the stability in the resistance to shearing and rotational forces.

## Materials and methods

A three-dimensional model of the Sawbones® left fourth-generation composite femur (Model 3406; Sawbones, Vashon, WA) was used for the geometric model of the femur.

**Fig. 1:**
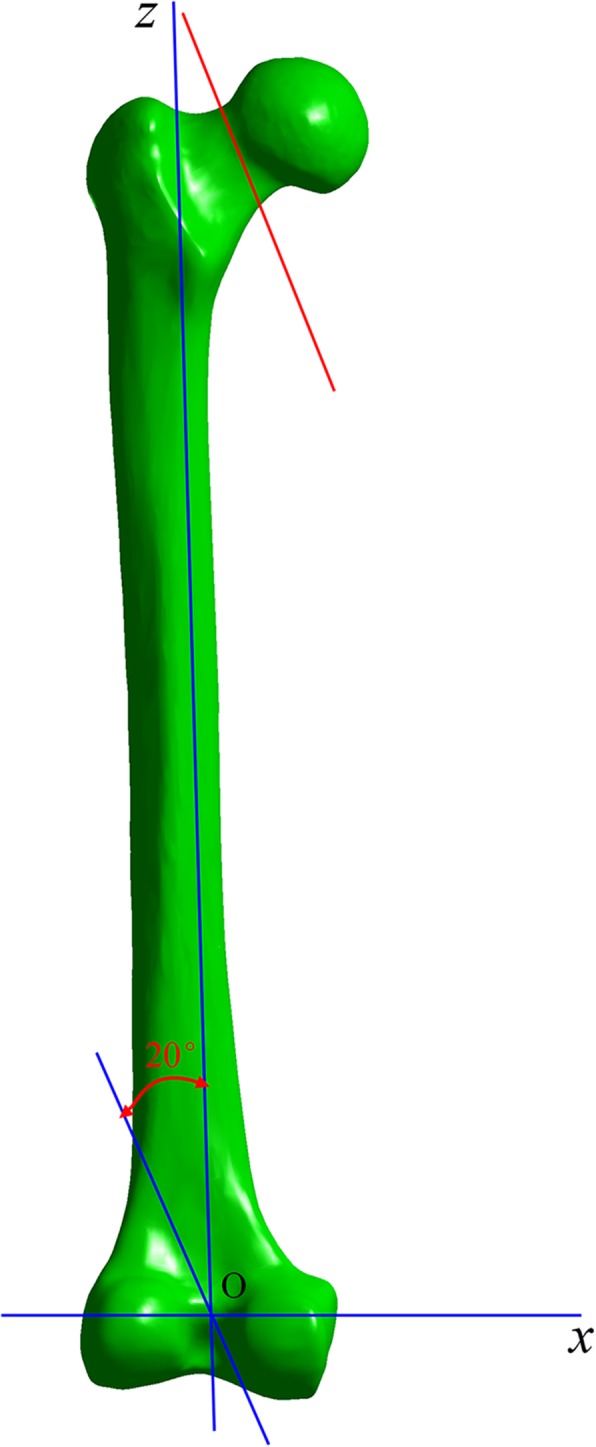
Femoral neck fracture line structure. The blue line ZO is the axis of the femoral shaft; the pink line is the fracture line of the femoral neck, and the angle of complement of this angle (20°) is the angle of the femoral neck fracture

Then, we used Solidworks software (Dassault, France) to simulate the Pauwels type III unstable fracture [11]. We first created the femoral shaft axis, a cross which a sagittal plane was created. Then, we created a cutting plate that was across the center of the femoral neck at an angle of 20° with respect to the sagittal plane of the shaft axis. The femoral neck was cut by the cutting plane, simulating a Pauwels type III fracture (Fig. 1).

**Fig. 2:**
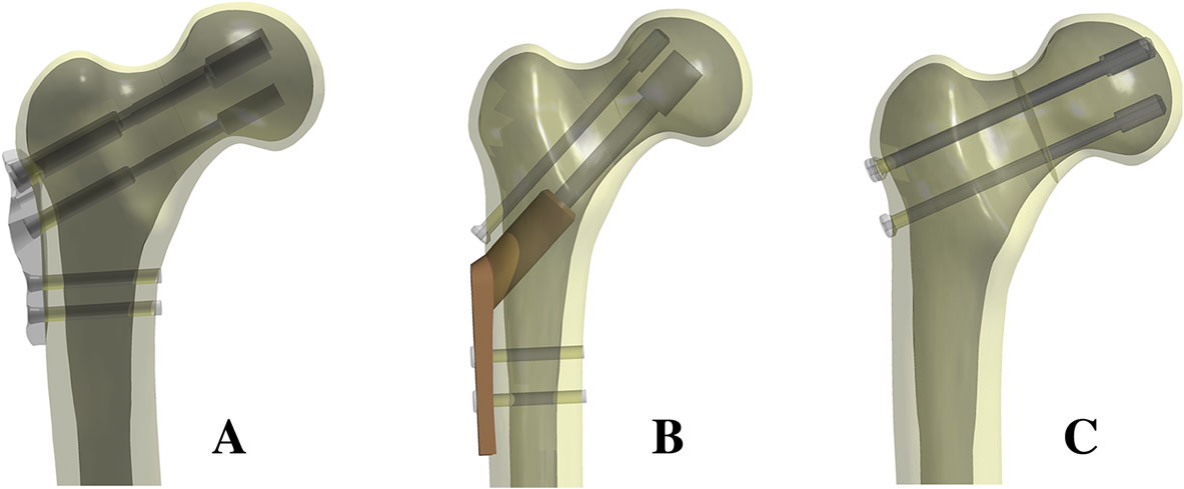
**a** SCAP-FN, **b** DHS+DS, **c** CCS. Geometric modeling of internal fixation of femoral neck fracture

According to clinical fixation programming and engineering geometric data modeling, we used Solidworks software to generate SCAP-FN (three sliding screws, each total length is 94 mm and 22 mm thread length, two distal full-thread locking screws are 44 mm length), DHS+DS (the lag screw is 20 mm threaded length and total length is 108 mm, two short screws are 41 mm in length, DS is 7.3 mm diameter and 16 mm thread length), and CCS (7.3 mm diameter and 16 mm thread length, each total length is 100 mm). Also, the femoral and internal fixation models were sequentially assembled, and all internal fixation screws were modeled in Solidworks. Specific models are shown in Fig. 2.

In ANSYS Workbench software (ANSYS, American), each assembly is meshed by solid 187 tetrahedral elements, and the grid convergence calculations are tested by different sizes. The statistics of three assembly elements and the total numbers of nodes are shown in Table 1.

**Table 1:** The statistics of three assembly units and the total amount of nodes

Case group	Node	Unit
SCAP	306,599	200,126
DHS+DS	303,444	202,846
CCS	220,032	142,647

About the material parameters, for modeling purposes, it was assumed that the cortical bone, cancellous bone, and femoral neck plate and locking screw were all continuous, isotropic, and uniform linear elastic materials. The list of parameters [12, 13] for each component used in the calculation is shown in Table 2.

**Table 2:** Bone and internal fixation material properties

Material name	Elastic modulus (MPa)	Poisson’s ratio
Cortical bone	16,350	0.26
Cancellous bone	137	0.3
Titanium alloy	110,000	0.3

According to the contact method described in references [12, 14], the fracture surface was set to friction (friction coefficient = 0.46). Frictional contact was used between the titanium plate and the bone surface (friction coefficient = 0.3); friction coefficients between the screw and sleeve = 0.23. The threaded screw area was used for binding to the bone, and the non-threaded area remained in contact with the bone. The screw was used to contact the titanium plate.

**Fig. 3:**
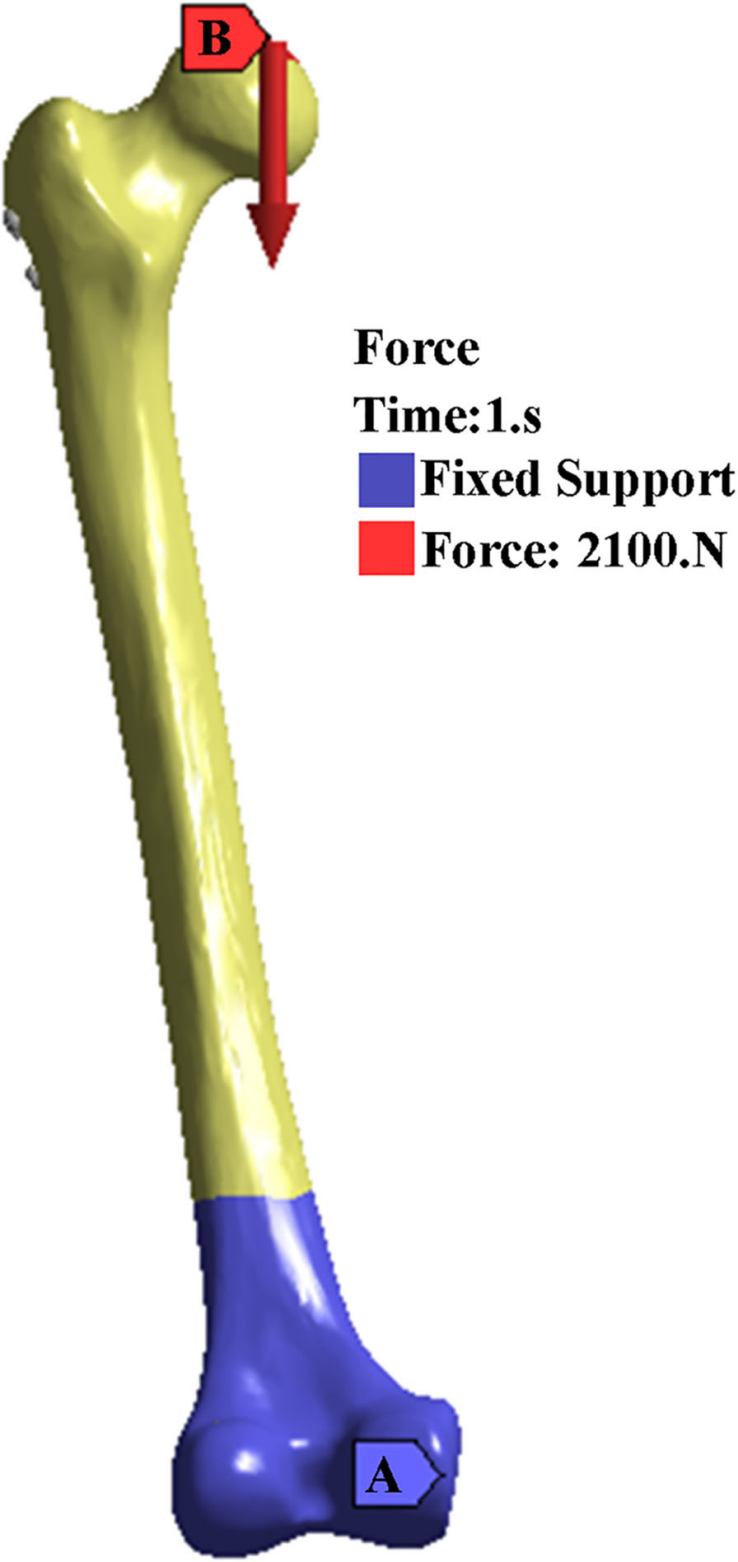
(**a**) The blue area means the distal femur is fixed. (**b**) the red arrow is the direction of the force

For calculation purposes, the distal end of the femur was completely fixed. To mimic the single-leg standing position [15], each calculated assembly model was abducted 10°, tilted backward by 9°, and statically loaded with a downward vertical force of 2100 N [16] (Fig. 3).

**Fig. 4:**
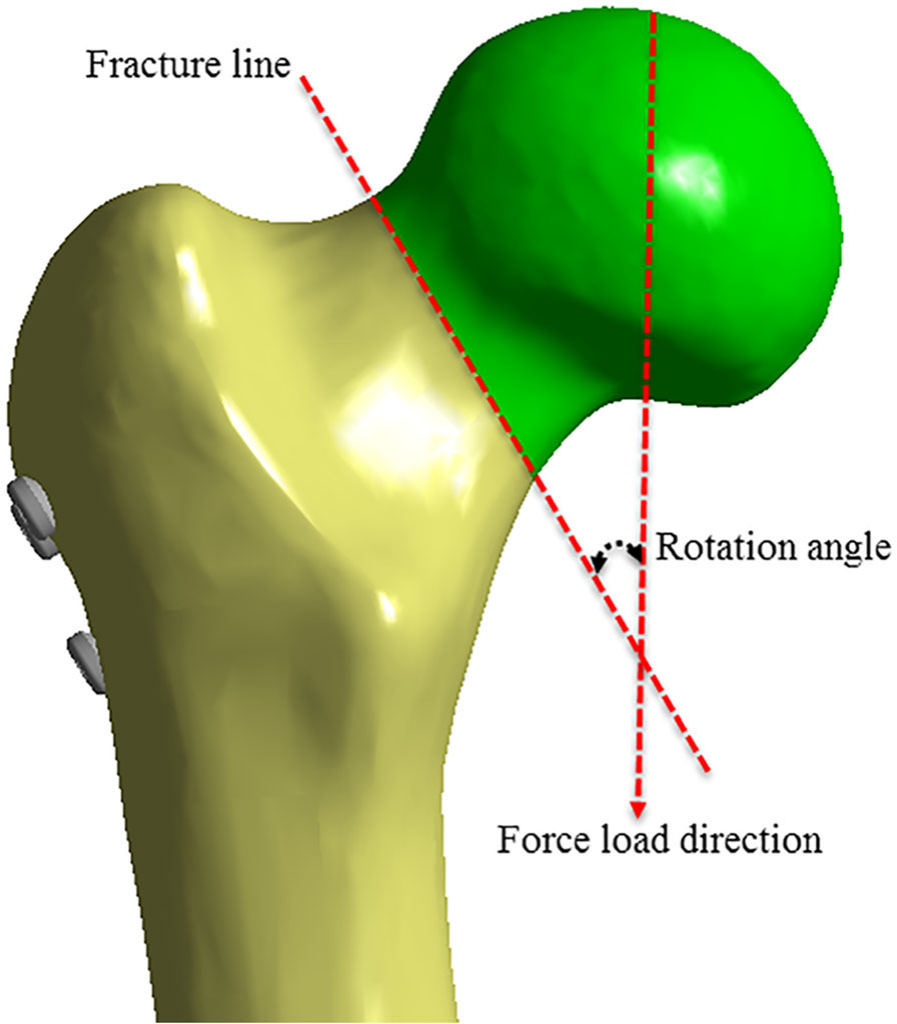
Rotation angle is the change of the angle before and after the external force is applied

An angle between the direction of the intended loading force and the direction of the fracture line was present for better comparison of the anti-rotation ability between the three internal fixations. As the direction of the vertical loading force is fixed, the direction of the fracture line varies according to the loading force. The angle between the sites then changes (referred to as the rotational angle) according to the loading force. This can be used to indirectly determine the anti-rotational stability of the internal fixation (Fig. 4).

## Results

**Fig. 5:**
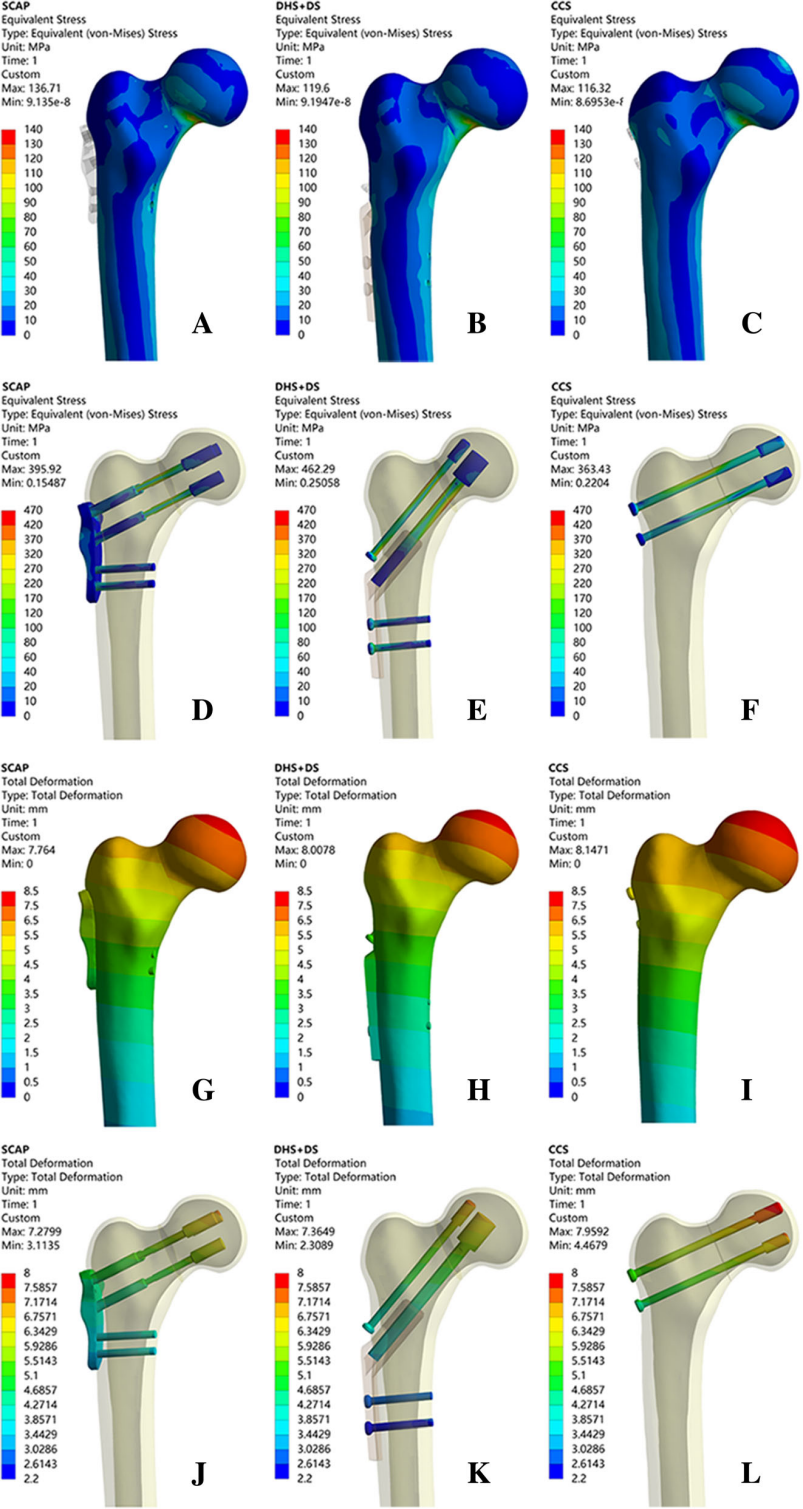
**a**–**c** The stress of femur. **d**–**f** The stress of internal fixation. **g**–**i** The displacement of the femur. **j**–**l** The displacement of the femur internal fixation

The femur and the stress distributions, stress peaks, and rotational angle of all three internal fixations were examined. Table 3 and Fig. 5 show the in detail the results.

**Table 3:** Parameters results

Parameters	SCAP	DHS+DS	CCS
The maximum displacement of the femur (mm)	7.764	8.0087	8.1479
The maximum displacement of the internal fixation (mm)	7.2799	7.3649	7.9592
Maximum femur stress (MPa)	136.71	119.6	116.32
Internal fixation maximum stress (MPa)	395.92	462.29	363.43
The rotation angle (°)	1.29	1.88	2.35

### Von Mises stress distribution

On the three configurations, differences of stress distribution were observed. In the femur, stresses appeared to be concentrated in the site a little above the fracture line of each group, and the peak Von Mises stresses at the femur were 116.32 MPa, 119.28 MPa, and 136.71 MPa in CCS, DHS+DS, and SCAP-FN, respectively.

In the three internal fixations, stresses appeared to be concentrated in the middle surface of the screw near the fracture line of each group, and the peak Von Mises stresses were 363.43 MPa, 461.25 MPa, and 395.92 MPa in CCS, DHS+DS, and SCAP-FN, respectively (Table 3 and Fig. 5).

### Displacement

According to the displacement contour of the femur, the maximum displacement occurs at the front of the femoral head, and the maximum displacements were 8.1479 mm, 8.0087 mm, and 7.764 mm in CCS, DHS+DS, and SCAP-FN, respectively.

According to the displacement contour of the internal fixation, the maximum displacement occurs at the head of the screws, and the maximum internal fixation displacements were 7.9592 mm, 7.3649 mm, and 7.2799 mm in CCS, DHS+DS, and SCAP-FN, respectively. These results demonstrate that the SCAP-FN group exhibited lower displacement compared to the other groups.

### Rotational angle

**Fig. 6:**
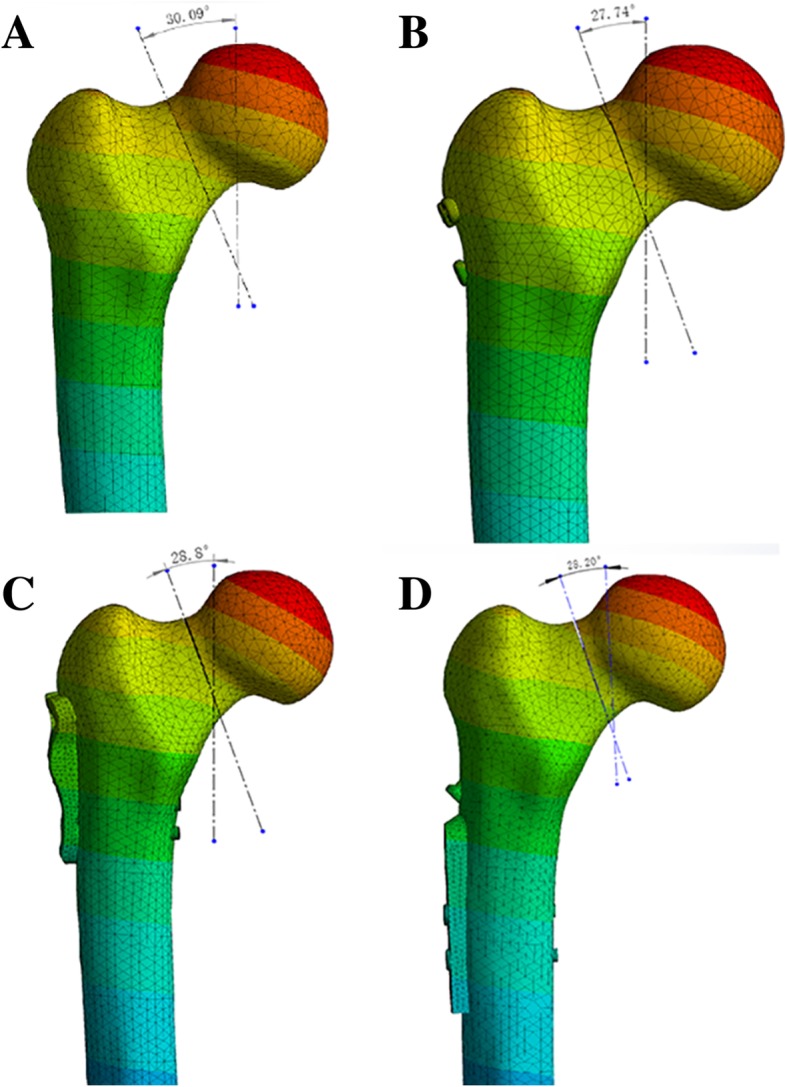
**a** The rotation angle the before stress loading. **b**–**d** The rotation angle after the stress loading in three groups

**Fig. 7:**
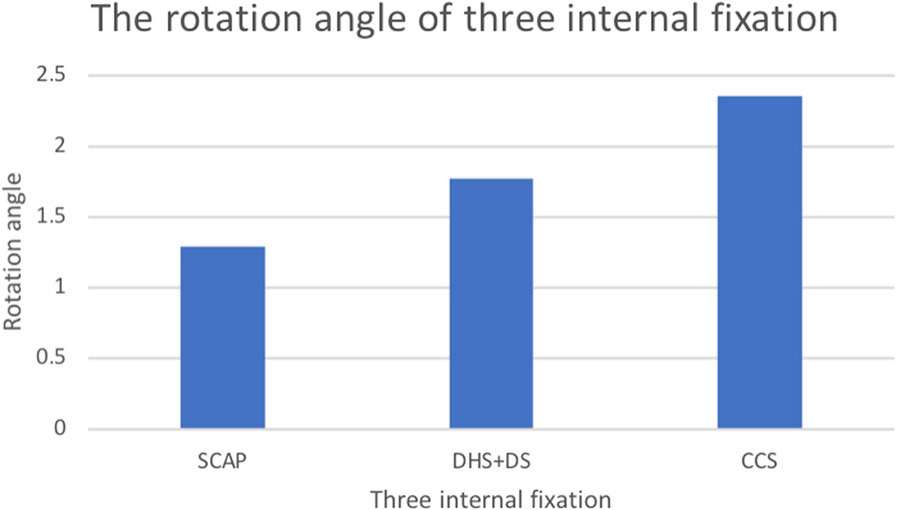
In this histogram, we can see that the rotation angle of SCAP is the smallest in the three groups

Before applying the loading force, the angle of the fracture line and direction of the loading force were 30.08°. After the test, the angles were 27.74°, 28.20°, and 28.8° in CCS, DHS+DS, and SCAP-FN, respectively. According to the change of angle, rotational angles were measured as 2.35°, 1.88°, and 1.29° in CCS, DHS+DS, and SCAP-FN, respectively. The rotational angle in SCAP was the smallest of the three groups (Fig. 6 and Fig. 7).

We have actually performed finite element analysis (FEA) by using real bones derived from young (33 years) and old patients (84 years), and they both showed consistent results with that by using Sawbone (Additional file 1: Figure S1 and Additional file 2: Figure S2; Additional file 3: Table S1 and Additional file 4: Table S2).

## Discussion

### Main findings

As expected, SCAP-FN retains the strength of both CCS and DHS+DS. It shows a good ability of resisting shearing and rotation force, therefore achieving the best stability in terms of smallest displacement and rotation angle.

The optimal approach to treat femoral neck fractures remains controversial. Arthroplasty is usually ruled out quickly, unless it is necessary, given that arthroplasty implants generally do not last more than 20 years and can cause multiple complications including infections and dislocations [4, 17]. Unfortunately, commonly used internal fixation strategies including cannulated screws and sliding hip screws often result in poor outcomes such as fixation failure and non-union. As understanding of underlying fracture mechanics has improved, clinicians have been able to better understand and explain these poor outcomes. The dominance of shearing and rotational force, in particular in the case of vertically displaced fractures, leads to femoral head toggling and rotation. It is thus vital that any fixation approach be capable of resisting these forces during the process of bone healing. Biomechanical experiments show that DHS has a slight improvement in shearing force resistance [18]. However, as only single screws are used, DHS has a lower ability to resist rotational force relative to the use of multiple cannulated screws [19, 20]. Consequently, patients treated with DHS are more likely to experience a rotational displacement of the femoral head. To overcome this weakness, an advanced approach that inserts a derotational screw (DS) in parallel to the main screw of DHS (DHS+DS) has been developed. Theoretically, the resistance to rotation is then enhanced, as confirmed in various biomechanical studies. However, in some clinical trials, no significant improvements in the outcome in terms of improved union rates and lower reoperation rates are observed. The reoperation rate for DHS+DS remained as high as 18% [21]. The underlying factors for this remain unclear.

Absolute stable internal fixation is beneficial to femoral neck fracture union; thus, the primary goal of internal fixation is to prevent displacement of fracture. An unstable femoral neck fracture adversely affects blood supply to the femoral head, which may lead to non-union and the necrosis of the femoral head. On the contrary, stabilization of the fracture through internal fixation allows revascularization to proceed in an optimal mechanical environment; therefore, higher stability is required in order to reduce the probability of non-union and avascular necrosis of the femoral head [22–24].

Our design retains the strength of a fixed-angle device with respect to resisting bending and shearing forces at the fracture site of the neck. In addition, three screws were inserted as a triangle configuration into the femoral neck and also attached to the lateral plate to prevent a rotational migration.

## Conclusion

The FEA encouraged us that in the following biomechanical experiment, SCAP-FN may remain the strengths of both CCS and DHS+DS and show a better performance in resisting shearing and rotational forces, therefore achieving the best stability in terms of the smallest displacement and rotational angle. Much as is the case for other FE analyses, studies assessing larger cohorts or randomized controlled studies (RCTs) including patients who require femoral neck fixation are needed in order to formally confirm our findings.

## Additional files

Additional file 1: **Figure S1.** Results for using young patient model. A-C. The stress of femur; D-F. The stress of internal fixation; G-I. The displacement of the femur; J-L. The displacement of the femur internal fixation. (TIF 5329 kb)

Additional file 2: **Figure S2.** Results for using old patient model. A-C. The stress of femur; D-F. The stress of internal fixation; G-I. The displacement of the femur; J-L. The displacement of the femur internal fixation. (TIF 6522 kb)

Additional file 3: **Table S1.** Finite element analyses on a young patient (33 years). (DOCX 13 kb)

Additional file 4: **Table S2.** Finite element analyses on an old patient (84 years). (DOCX 13 kb)

## Abbreviations

DS: Derotational screw
FEA: Finite element analysis
SCAP-FN: Slide compression anatomic place-femoral neck

## References

[1] Abrahamsen B, van Staa T, Ariely R, Olson M, Cooper C (2009). Excess mortality following hip fracture: a systematic epidemiological review. Osteoporos Int.

[2] Klop C, Welsing PMJ, Cooper C, Harvey NC, Elders PJM, Bijlsma JWJ (2014). Mortality in British hip fracture patients, 2000-2010: a population-based retrospective cohort study. Bone.

[3] Roche JJW, Wenn RT, Sahota O, Moran CG (2005). Effect of comorbidities and postoperative complications on mortality after hip fracture in elderly people: prospective observational cohort study. BMJ.

[4] Bhandari M, Devereaux PJ, Swiontkowski MF, Tornetta P, Obremskey W, Koval KJ (2003). Internal fixation compared with arthroplasty for displaced fractures of the femoral neck. A meta-analysis. J Bone Joint Surg Am.

[5] Mundi S, Pindiprolu B, Simunovic N, Bhandari M (2014). Similar mortality rates in hip fracture patients over the past 31 years. Acta Orthop.

[6] Kakar S, Tornetta P, Schemitsch EH, Swiontkowski MF, Koval K, Hanson BP (2007). Technical considerations in the operative management of femoral neck fractures in elderly patients: a multinational survey. J Trauma.

[7] Siavashi B, Aalirezaei A, Moosavi M, Golbakhsh MR, Savadkoohi D, Zehtab MJ (2015). A comparative study between multiple cannulated screws and dynamic hip screw for fixation of femoral neck fracture in adults. Int Orthop.

[8] Brandt E, Verdonschot N, van Vugt A, van Kampen A (2011). Biomechanical analysis of the sliding hip screw, cannulated screws and Targon® FN in intracapsular hip fractures in cadaver femora. Injury.

[9] Rupprecht M, Grossterlinden L, Ruecker AH, de Oliveira AN, Sellenschloh K, Nüchtern J (2011). A comparative biomechanical analysis of fixation devices for unstable femoral neck fractures: the Intertan versus cannulated screws or a dynamic hip screw. J Trauma Injury Infection Critical Care.

[10] Prof Mohit Bhandari. Fracture fixation in the operative management of hip fractures (FAITH): an international, multicentre, randomised controlled trial. Lancet. 2017 15;389(10078):1519–27. 10.1016/S0140-6736(17)30066-1 PMC5597430 28262269

[11] Rupprecht M, Grossterlinden L, Ruecker AH, de Oliveira AN, Sellenschloh K, Nüchtern J (2011). A comparative biomechanical analysis of fixation devices for unstable femoral neck fractures: the Intertan versus cannulated screws or a dynamic hip screw. J Trauma.

[12] Samsami S, Saberi S, Sadighi S, Rouhi G (2015). Comparison of three fixation methods for femoral neck fracture in young adults: experimental and numerical investigations. J Med Biol Eng.

[13] MacLeod AR, Rose H, Gill HS (2016). A validated open-source multisolver fourth-generation composite femur model. J Biomech Eng.

[14] Chen WP, Tai CL, Shih CH, Hsieh PH, Leou MC (2004). Selection of fixation devices in proximal femur rotational osteotomy: clinical complications and finite element analysis. Clin Biomech (Bristol, Avon).

[15] Hunt S, Martin R, Woolridge B (2012). Fatigue testing of a new locking plate for hip fractures. J Med Biol Eng..

[16] Zhang Y, Tian L, Yan Y, Sang H, Ma Z, Jie Q (2011). Biomechanical evaluation of the expansive cannulated screw for fixation of femoral neck fractures. Injury.

[17] Alolabi B, Bajammal S, Shirali J, Karanicolas PJ, Gafni A, Bhandari M (2009). Treatment of displaced femoral neck fractures in the elderly: a cost-benefit analysis. J Orthop Trauma.

[18] Aminian A, Gao F, Fedoriw WW, Zhang L-Q, Kalainov DM, Merk BR (2007). Vertically oriented femoral neck fractures: mechanical analysis of four fixation techniques. J Orthop Trauma.

[19] Chen Z, Wang G, Lin J, Yang T, Fang Y, Liu L (2011). Efficacy comparison between dynamic hip screw combined with anti-rotation screw and cannulated screw in treating femoral neck fractures. Zhongguo Xiu Fu Chong Jian Wai Ke Za Zhi.

[20] Zhang LL, Zhang Y, Ma X, Liu Y (2017). Multiple cannulated screws vs. dynamic hip screws for femoral neck fractures: a meta-analysis. Orthopade.

[21] Enocson A, Lapidus LJ (2012). The vertical hip fracture - a treatment challenge. A cohort study with an up to 9 year follow-up of 137 consecutive hips treated with sliding hip screw and antirotation screw. BMC Musculoskelet Disord.

[22] Swiontkowski MF (1994). Intracapsular fractures of the hip. J Bone Joint Surg Am.

[23] Rodríguez-Merchán EC (2002). In situ fixation of nondisplaced intracapsular fractures of the proximal femur. Clin Orthop Relat Res.

[24] Bjørgul K, Reikerås O (2007). Outcome of undisplaced and moderately displaced femoral neck fractures. Acta Orthop.

