# Correction to: Comparison of three different internal fixation implants in treatment of femoral neck fracture—a finite element analysis

**DOI:** 10.1186/s13018-019-1148-3

**Published:** 2019-04-16

**Authors:** Jia Li, Zhe Zhao, Pengbin Yin, Licheng Zhang, Peifu Tang

**Affiliations:** 1 Department of Orthopedics, Chinese PLA General Hospital, No. 28 Fuxing Road, Beijing, 100853 People’s Republic of China; 2 Department of Orthopaedics, Beijing Tsinghua Changgung Hospital, School of Clinical Medicine, Tsinghua University, No. 168, Li Tang Road, Changping District, Beijing, 102218 China

**Correction to: J Orthop Surg Res**

**https://doi.org/10.1186/s13018-019-1097-x**

In the original publication of this article [1], the word “place” should be changed to “plate”. This word occurs at four sections: 1. Methods of the Abstract; 2. Keywords of the Abstract; 3 Introduction; 4. Abbreviations.
