# Supplementary figures and images for: Comparison of three different internal fixation implants in treatment of femoral neck fracture—a finite element analysis
Source: J Orthop Surg Res. 2019 Mar 12;14:76. doi: 10.1186/s13018-019-1097-x (PMC6419341; doi:10.1186/s13018-019-1097-x)

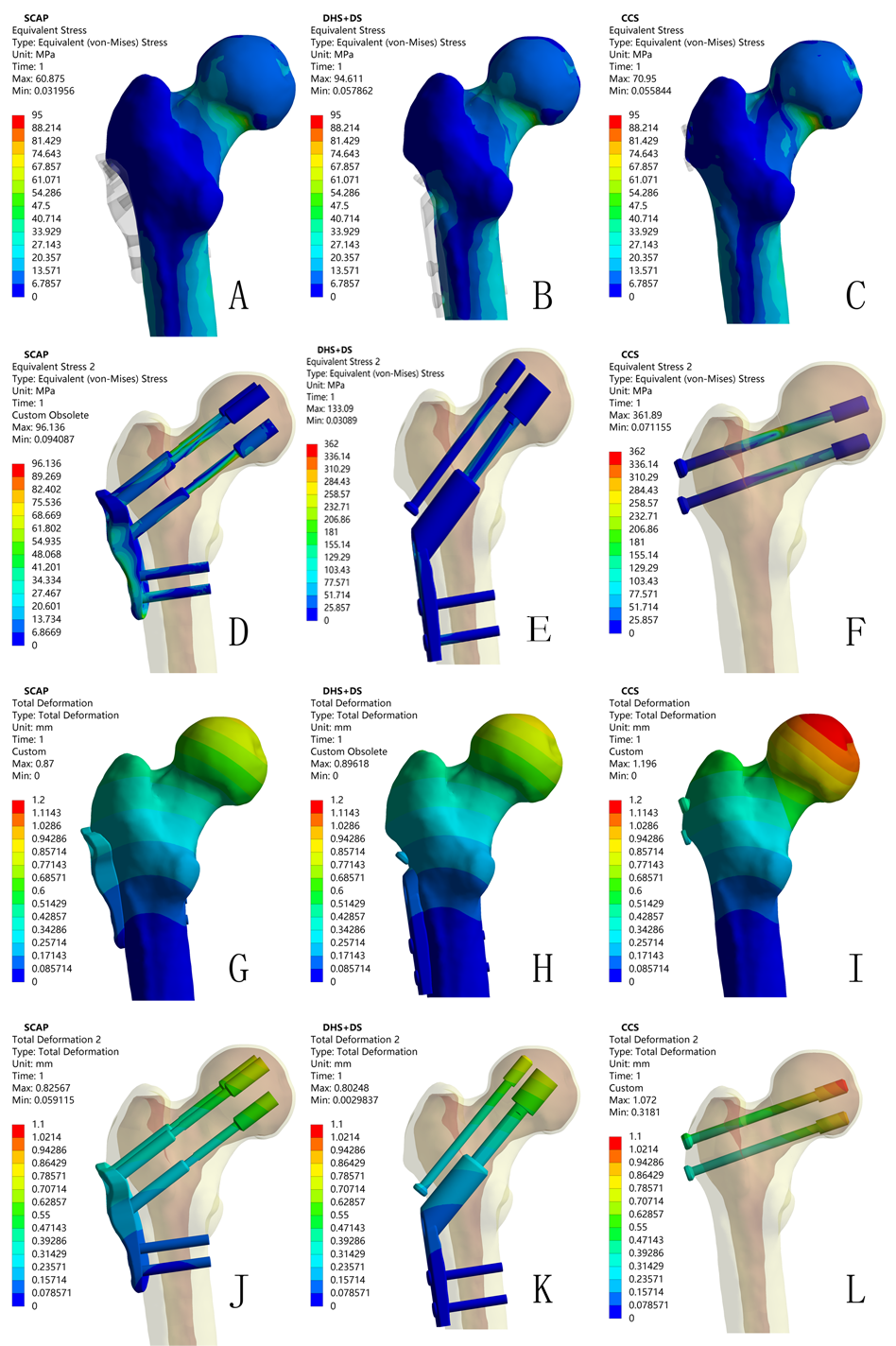

Supplement: Supplementary file 1 — Figure S1. Results for using young patient model. A-C. The stress of femur; D-F. The stress of internal fixation; G-I. The displacement of the femur; J-L. The displacement of the femur internal fixation. (TIF 5329 kb) [file 13018_2019_1097_MOESM1_ESM.tif]

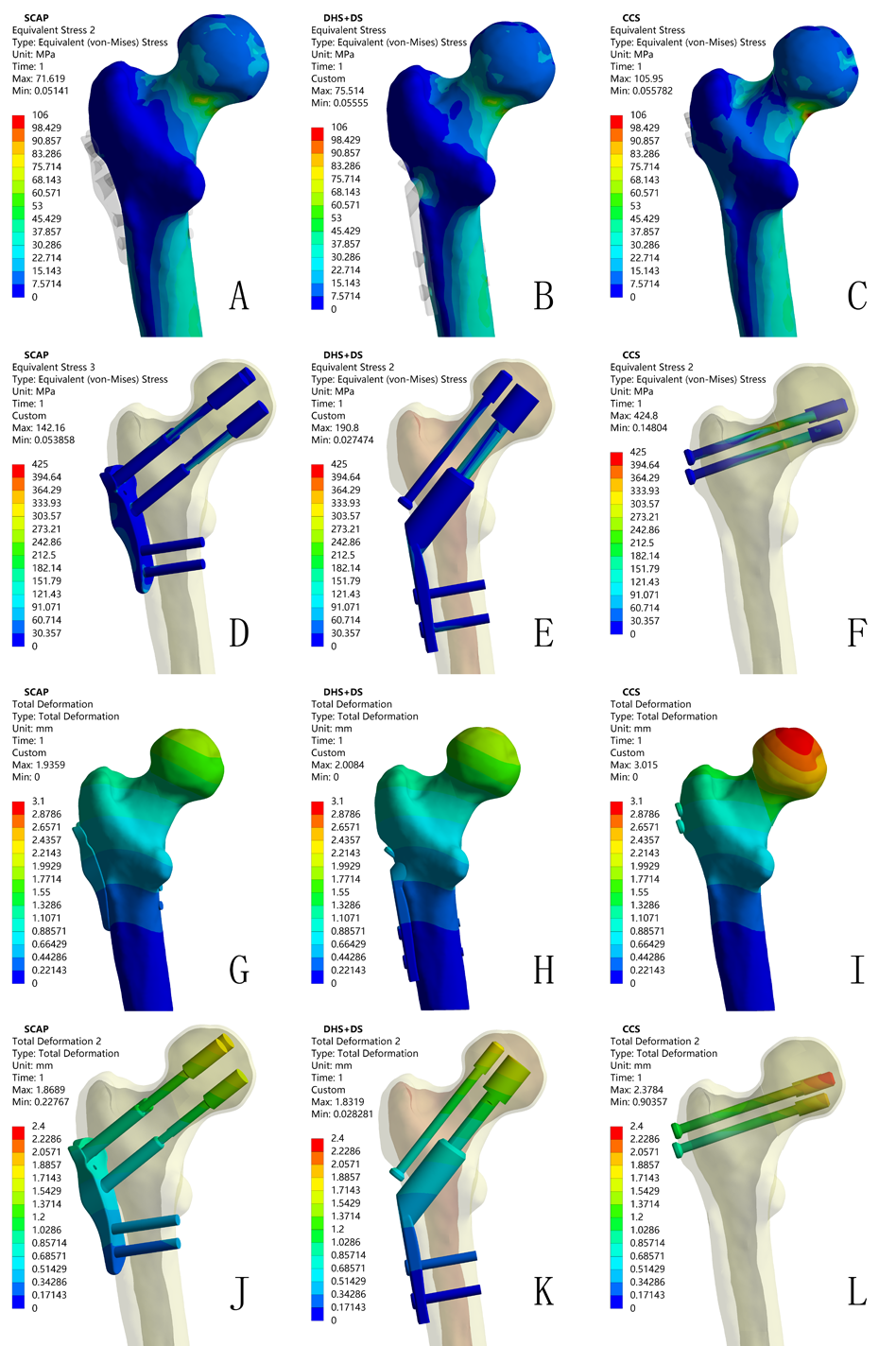

Supplement: Supplementary file 2 — Figure S2. Results for using old patient model. A-C. The stress of femur; D-F. The stress of internal fixation; G-I. The displacement of the femur; J-L. The displacement of the femur internal fixation. (TIF 6522 kb) [file 13018_2019_1097_MOESM2_ESM.tif]
