# Supplementary material for: Comparison of three different internal fixation implants in treatment of femoral neck fracture—a finite element analysis
Source: J Orthop Surg Res. 2019 Mar 12;14:76. doi: 10.1186/s13018-019-1097-x (PMC6419341; doi:10.1186/s13018-019-1097-x)
Supplement: Supplementary file 3 — Table S1. Finite element analyses on a young patient (33 years). (DOCX 13 kb) [file 13018_2019_1097_MOESM3_ESM.docx]

Table S1 Finite element analyses on a young patient (33y)

| Parameters | SCAP | DHS+DS | CCS |
| --- | --- | --- | --- |
| The maximum displacement of the femur (mm) | 0.87 | 0.89618 | 1.196 |
| The maximum displacement of the Internal fixation (mm) | 0.82567 | 0.80248 | 1.072 |
| Maximum femur stress (MPa) | 60.875 | 94.611 | 70.95 |
| Internal fixation maximum stress (MPa) | 96.136 | 133.09 | 361.89 |
| The rotation angle(°) | 0.353333 | 0.393333 | 0.53333 |
