# Supplementary material for: Comparison of three different internal fixation implants in treatment of femoral neck fracture—a finite element analysis
Source: J Orthop Surg Res. 2019 Mar 12;14:76. doi: 10.1186/s13018-019-1097-x (PMC6419341; doi:10.1186/s13018-019-1097-x)
Supplement: Supplementary file 4 — Table S2. Finite element analyses on an old patient (84 years). (DOCX 13 kb) [file 13018_2019_1097_MOESM4_ESM.docx]

Table S2 Finite element analyses on an old patient (84y)

| Parameters | SCAP | DHS+DS | CCS |
| --- | --- | --- | --- |
| The maximum displacement of the femur (mm) | 1.9359 | 2.0084 | 1.196 |
| The maximum displacement of the Internal fixation (mm) | 1.8689 | 1.8319 | 1.072 |
| Maximum femur stress (MPa) | 71.619 | 75.514 | 70.95 |
| Internal fixation maximum stress (MPa) | 142.16 | 190.8 | 361.89 |
| The rotation angle(°) | 0.35 | 0.91 | 1.91 |
